# Contributions of Molecular and Optical Techniques to the Clinical Diagnosis of Alzheimer’s Disease

**DOI:** 10.3390/brainsci10110815

**Published:** 2020-11-03

**Authors:** Edoardo Bistaffa, Fabrizio Tagliavini, Paolo Matteini, Fabio Moda

**Affiliations:** 1 Fondazione IRCCS Istituto Neurologico Carlo Besta, Division of Neurology 5 and Neuropathology, 20133 Milan, Italy; edoardo.bistaffa@istituto-besta.it; 2 Fondazione IRCCS Istituto Neurologico Carlo Besta, Scientific Directorate, 20133 Milan, Italy; fabrizio.tagliavini@istituto-besta.it; 3 IFAC-CNR, Institute of Applied Physics “Nello Carrara”, National Research Council, 50019 Sesto Fiorentino, Italy

**Keywords:** Alzheimer’s disease, optical techniques, protein misfolding, RT-QuIC, clinical diagnosis, surface-enhanced Raman spectroscopy

## Abstract

Alzheimer’s disease (AD) is the most common neurodegenerative disorder worldwide. The distinctive neuropathological feature of AD is the intracerebral accumulation of two abnormally folded proteins: β-amyloid (Aβ) in the form of extracellular plaques, and tau in the form of intracellular neurofibrillary tangles. These proteins are considered disease-specific biomarkers, and the definite diagnosis of AD relies on their post-mortem identification in the brain. The clinical diagnosis of AD is challenging, especially in the early stages. The disease is highly heterogeneous in terms of clinical presentation and neuropathological features. This phenotypic variability seems to be partially due to the presence of distinct Aβ conformers, referred to as strains. With the development of an innovative technique named Real-Time Quaking-Induced Conversion (RT-QuIC), traces of Aβ strains were found in the cerebrospinal fluid of AD patients. Emerging evidence suggests that different conformers may transmit their strain signature to the RT-QuIC reaction products. In this review, we describe the current challenges for the clinical diagnosis of AD and describe how the RT-QuIC products could be analyzed by a surface-enhanced Raman spectroscopy (SERS)-based systems to reveal the presence of strain signatures, eventually leading to early diagnosis of AD with the recognition of individual disease phenotype.

## 1. Alzheimer’s Disease is a Double-Prion Disorder

Alzheimer’s disease (AD) is the most frequent neurodegenerative disorder in elderly people affecting nowadays, about 44 million patients worldwide, and this estimation is expected to double every 20 years [1]. For this reason, AD represents one of the great health-care challenges of the 21st century, but from the discovery of the key component of AD pathogenic mechanism, little is known about the causes of the disease, and no effective treatments have been developed so far. Most cases have a sporadic origin and occur in subjects over the age of 65, while less than 5% of all the cases occur earlier [2]. Mutations in the amyloid precursor protein (APP), presenilin-1 (PSEN1), and presenilin-2 (PSEN2) are known to cause familial forms of AD that are characterized by early onset and rapid progression [3,4,5,6,7,8]. The apolipoprotein E gene (ApoE) represents a genetic risk factor for AD. It has three common alleles: ɛ2, ɛ3, and ɛ4 and the allelic variant ɛ4/ɛ4 is associated with high susceptibility to AD in the general population [9]. AD leads inevitably to dementia with progressive impairment of physiological functions, and the death generally occurs within 5–12 years from symptoms onset [10]. The main neuropathological hallmark of AD is the aggregation in the brain of two different proteins: (1) β-amyloid (Aβ), which deposits extracellularly to form amyloid plaques, and (2) tau, which accumulates intraneuronally to form neurofibrillary tangles (NFTs) made up of hyperphosphorylated-tau. Before aggregating, both Aβ and tau undergo a process of structural rearrangement (*misfolding*), resulting in a higher content of β-sheet in their secondary structures [11]. Aβ plaques and NFTs are considered specific biomarkers for AD, and their deposition often parallel the cognitive decline [12]. Emerging evidence suggests that both Aβ and tau proteins possess prion-like features [13,14,15]. What does it mean? In 1982 the Nobel prize winner, prof. Stanley Prusiner [16], discovered that an infectious protein was responsible for a group of neurodegenerative disorders, named prion diseases. This discovery has vexed the scientific community because it was against the central dogma of molecular biology [17]. Indeed, in this case, a single protein with aberrant conformations (*misfolded*) was able to cause disease. Prof. Prusiner coined the term ‘prion’ (proteinaceous infectious only particle) to refer to this misfolded protein that was characterized by infectious and pathological properties, and that was able to ‘replicate’ even without the presence of genetic material. In particular, prion (or PrP^Sc^) arises from the conformational conversion of the normal cellular prion protein (PrP^C^), which is expressed in many tissues, including the central nervous system (CNS) [18]. Several studies have confirmed that PrP^Sc^ alone can cause disease and sustain its progression. Once generated, PrP^Sc^ coerces other PrP^C^ to misfold, thus, generating new PrP^Sc^ molecules by overcoming the thermodynamic barriers which normally prevent this phenomenon from occurring [19]. In this way, PrP^Sc^ spreads throughout the brain and aggregates to form oligomers, which then assemble into insoluble amyloid fibrils. One of the most fascinating aspects of these diseases is that PrP^Sc^ can acquire distinct abnormal conformations, referred to as *strains*, which are responsible for at least six different phenotypes of prion disorders [20,21]. Each prion strain possesses peculiar properties (in terms of pathological and biochemical features), which confer a distinctive *signature* useful for their identification. Since prions replicate without the presence of genetic instructions, the abnormal structure is believed to encipher and propagate individual prion strain signature [22]. This represents an innovative and unusual biological paradigm of information storage and transmission. The prion paradigm is now expanding to AD and some other neurodegenerative disorders characterized by the presence of misfolded proteins, including Parkinson’s disease (PD), Huntington’s disease (HD), and Amyotrophic Lateral Sclerosis (ALS) [23,24,25,26]. In AD, Aβ and tau undergo misfolding and acquire distinct aberrant structures that significantly influence their interactome, their tropism for defined neuronal cells, and their capacity to modulate specific cellular pathways. Once misfolded, both proteins can template the conformational conversion of their normal counterparts and propagate in the brain following defined spatiotemporal patterns [27,28]. For this reason, they are considered prion-like proteins capable of sustaining disease progression [29,30,31], and AD can be thought of as a double prion disorder. AD is phenotypically heterogeneous, and this variability might be sustained, at least in part, by a spectrum of conformationally diverse Aβ aggregates, referred to as Aβ *strains*. Thus, similarly to prion diseases, Aβ seems to have important roles in disease pathogenesis and is included among the debated causal factors of AD, as well as the loss of cholinergic neurons, the deregulation of neuronal cell cycle, and the presence of neuroinflammatory molecules and toxic products [32,33,34,35,36,37,38]. Nevertheless, a growing body of evidence sustains that different strains of Aβ are believed to be responsible for different phenotypes of AD. Several factors, including chaperones, inflammatory cytokines, ApoE, microbiota, lifestyle, and other elements still unknown, could contribute to disease variability, especially by influencing the interactions between misfolded Aβ and other cellular components (as well as tau) [39,40,41,42,43,44,45,46,47,48,49].

Recent studies, including electron cryo-microscopy (cryo-EM) and immuno-gold electron microscopy (immuno-EM) analyses, showed that the abnormal conformations of tau between patients with sporadic and genetic forms of AD are not that different. Probably, the intracellular environment limits the range of structural rearrangements adoptable by the protein [50]. In contrast, Aβ aggregates extracellularly, in a different environment, which is likely more permissive to the generation of multiple Aβ strains that can be key determinants of disease heterogeneity. Therefore, at present, there is a strong correlation between the phenotypic heterogeneity of AD and Aβ strains.

## 2. Phenotypic Heterogeneity of Alzheimer’s Disease and the Role of Aβ Strains

The exact mechanisms through which Aβ misfolds are still not well understood. Nevertheless, it is known that Aβ can adopt a variety of abnormal conformations (Aβ *strains*), which are believed to account for the phenotypic heterogeneity of AD (in terms of clinical presentation, neuropathological features, and neuroanatomical involvement) [51,52,53]. Several studies have shown that among AD patients, it is possible to identify different types of Aβ deposits characterized by distinct morphology and biochemical features [52,54,55,56,57,58]. These findings strengthen the causal link between the Aβ structures and disease phenotype, but also highlight the possibility that more than one Aβ strain can be present in the same brain, while contributing to disease heterogeneity [59]. A variety of in vivo studies performed using transgenic mice reinforced this concept [51,53,60,61,62,63]. For instance, inoculations of susceptible animals with different Aβ aggregates, either synthetically produced or present in brain homogenates of patients with distinct AD phenotypes, led to cerebral Aβ deposition characterized by different morphology and distribution [45,52,53,61,64,65,66]. In some cases, the types of Aβ aggregates found in animals faithfully reproduced the neuropathological changes found in the brain of the AD donor [62]. All these findings suggest that every Aβ strain tries to transmit its own *signature* to normal Aβ, either during its pathological spreading in the brain of the patient or when inoculated in susceptible mice. Indeed, as for prions, Aβ strains can interact with the normal Aβ and coerce it to acquire similar abnormal structures, thus, transmitting individual strain *signature* [52,67]. In 2014, Stohr and colleagues [63] showed that two distinct misfolded Aβ conformers prepared using synthetic peptides composed of either 40 (Aβ_40_) or 42 (Aβ_42_) residues were able to generate distinct amyloid pathology in susceptible mice, thus, confirming that the pathological properties of each conformer were enciphered in the structure. A better comprehension of the molecular events which lead to the generation of distinct phenotypes of AD is becoming extremely important, especially in consideration of the fact that they might differentially respond to pharmacological treatments. But, above all, the recognition of the individual disease phenotype in patients in the early stages of AD will consent to develop tailored treatments. 

Whether Aβ strains are *bona fide* prions is still a debated issue. In any case, thanks to the prion-like behavior of Aβ, it is now possible to study the process of Aβ protein misfolding in vitro by exploiting an innovative technology developed in the field of prion diseases named Real-Time Quaking-Induced Conversion (RT-QuIC) [68]. This technique is useful to study Aβ misfolding, but also the factors that could drive and influence its structural rearrangement. Finally, it represents a new ultrasensitive platform potentially exploitable for the clinical diagnosis of AD (see below) [69].

## 3. Challenges in the Clinical Diagnosis of Alzheimer’s Disease 

AD progresses toward three stages: An early preclinical stage where no symptoms are observed (although the process of Aβ and tau misfolding, spreading, and aggregation are already in progress); a middle stage characterized by the presence of mild cognitive impairment (MCI) where memory and thinking functions are impaired (without significant interferences with the normal life of the patient); and a late-stage marked by the presence of many symptoms of dementia (memory loss, visual/spatial problems, the patient is no longer independent). MCI not always leads to AD, but can evolve to other forms of dementia, including frontotemporal dementia (FTD) or dementia with Lewy bodies (DLB), or can even revert back to a normal condition [70,71,72]. The main challenge in the field of AD is represented by the impossibility to formulate the correct diagnosis of the disease in its early stages when potential treatments can be provided before extensive brain damages have occurred. At present, the diagnosis of AD is made following criteria that rely on clinical, neuropsychological, instrumental (e.g., magnetic resonance imaging, fludeoxyglucose F 18 positron emission tomography—PET, amyloid PET, tau PET, and computed tomography) and laboratory tests [40]. The first criteria for the diagnosis of AD were defined in 1984 by the National Institute of Neurological and Communicative Disorders and Stroke (NINCDS) and the Alzheimer’s Disease and Related Disorders Association (ADRDA) [73]. In 2011, these criteria were revised and updated by the National Institute on Aging–Alzheimer’s Association (NIA-AA) and classified AD as ‘possible’ (the patient shows predominant amnestic dementia with an atypical course that can be due to other causes) or ‘probable’ (the patient shows predominant amnestic dementia with an insidious onset and history of progressive worsening, without other causes identified) [40,74]. At present, the in vivo diagnostic accuracy for AD is about 77%, even among expert neurologists [75]. At least in part, this uncertainty is because the diagnostic criteria indicate to analyze biomarkers that can be altered in AD, but also in some other conditions. For this reason, ‘proven’ (definitive) diagnosis of AD can be achieved only at post-mortem, after extensive neuropathological analyses aimed at identifying the presence of Aβ and NFTs aggregates in the brain. As previously mentioned, details about the localization and the biochemical/physicochemical features of Aβ (its *signature*) is important to identify the phenotypes of AD. Unfortunately, the recognition of these pathological variants is not possible when patients are alive. Therefore, there is an urgent need to uncover more sensitive biomarkers for early diagnosis and recognition of AD phenotypes. Recent evidence suggests that misfolded Aβ and tau do not remain confined to the brain, but can spread, in traces, to other tissues, including the cerebrospinal fluid (CSF). It is important to take advantage of these new findings to develop innovative diagnostic approaches that can identify both peripheral AD biomarkers for improving the clinical diagnostic accuracy of AD.

## 4. Alzheimer’s Disease Biomarkers: The Role of CSF and Other Peripheral Tissues

Nowadays, CSF biomarkers have been shown to have a high diagnostic performance to identify AD patients [76]. CSF can be collected through a lumbar puncture, and its alterations reflect brain pathophysiology [77]. In particular, several CSF biomarkers are investigated for evaluating the main neuropathological changes of AD: (1) Aβ deposition, (2) tau deposition (NFTs), and (3) neuronal loss. Among them, the peptide composed of 42 amino acids (Aβ_1-42_) is decreased in the CSF of AD patients. This reduction is associated with an increased concentration of the peptide in the brain, in the form of extracellular Aβ_1-42_ amyloid plaques [78,79].

PET imaging studies with an amyloid-binding agent (Pittsburgh Compound-B, PIB) strengthen the inverse correlation between Aβ_1-42_ levels in the brain and CSF. In particular, high concentrations of Aβ_1-42_ in the brain of AD patients were paralleled by low levels in the CSF [80,81]. There are subjects known to hyper produce Aβ proteins, and this might hamper the possibility to observe a real reduction of Aβ_1-42_ in their CSF samples. For this reason, the Aβ peptide composed of 40 amino acids (Aβ_1-40_) has been introduced among the CSF biomarkers to limit this potential issue. The concentration of Aβ_1-40_ in the CSF does not change in patients with AD [82,83]. Therefore, the ratio between Aβ_1-42_/Aβ_1-40_ consents to identify a real reduction of Aβ_1-42_ in the CSF of AD patients and can identify AD from other non-AD dementias with 85% sensitivity and 82% specificity [84,85,86]. Moreover, this indicator well correlates with the amyloid PET results [87,88].

Another important CSF biomarker for AD is the tau protein (T-tau) and its phosphorylated form at threonine 181 (P-tau). During disease progression, as a result of neuronal death, T-tau and P-tau are released in the CSF of AD patients, and their concentrations significantly increase. However, T-tau proteins increase also in the CSF of patients with other neurodegenerative diseases characterized by severe neuronal loss, including among the others, prion disorders (e.g., Creutzfeldt-Jakob disease). Therefore, this marker is not specific for AD even though it well correlates with the cognitive decline in AD patients [89,90,91,92]. In contrast, increased P-tau levels are more specific for AD and generally do not increase in other degenerative conditions [93,94,95]. A lot of work has been done, and is currently in progress, to update, standardize and harmonize the pre-analytical, analytical, and post-analytical procedures related to CSF biomarkers collection and analysis. Several consortia, including the Biomarkers for AD and Parkinson’s Disease (BIOMARKAPD), the Global Biomarkers Standardization Consortium (GBSC), the AD Neuroimaging Initiative (ADNI), the Alzheimer Biomarker Standardization Initiative (ABSI), and the JPND BIOMARKPD run huge efforts to ensure reproducible and consistent CSF biomarkers measurements for facilitating worldwide comparison of the results and maximizing their diagnostic utility [96,97,98]. The analysis of Aβ_1–42_ (or the ratio Aβ_1–42_/Aβ_1–40_) T-tau, and P-tau is currently highly valuable to discriminate between AD and non-AD patients, as well as cognitively healthy controls [99]. However, new potential biomarkers for AD present in the CSF, but also blood and urine are currently under investigation. For instance, the S100B protein was found to be significantly higher in the CSF of AD and FTD patients compared to controls [100]. A very recent publication indicates that tau phosphorylated at residue 217 (P-tau_217_) seems to perform better than the classical P-tau to identify patients with AD and might represent a better biomarker for the clinical diagnosis of the disease [101]. Remarkably, several studies showed that most of tau in CSF of AD patients is present as fragments. In this regard, the novel N-terminal tau fragments ending at amino acid 224 (N-224) were found to be significantly higher in AD patients compared to controls and correlated to cognitive impairment over time [102].

The neurofilament light chain protein (NfL) was also proposed as an innovative CSF biomarker for predicting brain atrophy, cognition, and Aβ accumulation in AD. In particular, the elevated levels of NfL in CSF correlate with white matter changes and axonal degeneration [103]. Moreover, the increase of NfL in CSF predicts hippocampal atrophy in cognitively healthy subjects at risk for AD [104] and correlate with disease progression [105]. Another protein that is currently being investigated as a new potential biomarker for AD is the neurogranin [106,107]. This protein is increased in the CSF of AD patients and correlates with the level of T-tau [108]. Moreover, an inverse correlation between neurogranin and Aβ_1–42_/Aβ_1–40_ ratio has been observed in the CSF of AD patients [108]. Finally, it seems that the ratio between neurogranin and the protein BACE1 is a potential correlate of cognitive decline in patients with AD [109]. The European Union-North American Clinical Trials in Alzheimer’s Disease Task Force (EU/US CTAD Task Force) has recently evaluated the status of blood-based AD biomarker and confirmed that (1) plasma Aβ levels accurately estimates the brain amyloid deposition, (2) plasma NfL levels correlates with neurodegeneration (although not specific for AD), and (3) plasma T-tau levels do not correlate with those of the CSF [110]. Thanks to the use of an innovative method based on immunoprecipitation coupled with mass spectrometry (IP-MS), it was demonstrated a reduction of Aβ_1-42_/Aβ_1-40_ ratio in plasma of AD patients versus control with a diagnostic accuracy of about 90% [111,112]. Similarly, the concentration of plasma tau was found to be higher in AD patients compared to healthy individuals [82,113,114,115]. Another study showed the levels of plasmatic P-tau_181_ were higher in AD patients than controls, and they correlated with disease severity [116]. Many other studies (including metabolomics approaches, miRNA and exosomes analyses) are currently ongoing to evaluate the diagnostic significance of blood-based or even urine-based AD biomarkers, including the last described proteins SPP1, GSN, and IGFBP7 that were found to be differentially expressed in the urine of AD patients compared to healthy subjects [117,118,119,120,121,122]. However, compared to CSF, these tissues have completely different matrix components, in terms of proteins (especially those binding to Aβ and tau) and factors, that might affect the efficient identification of AD biomarkers. Moreover, their alterations can be identified during the MCI stage, thus, limiting the possibility to identify AD patients in the very early stages of the disease [76].

With the development of highly sensitive techniques, traces of Aβ strains were found in the CSF of patients with AD [123]. Certainly, the identification of misfolded Aβ in this tissue will consent to formulate a considerably more accurate diagnosis of AD. Based on this premise, we decided to set-up an ultrasensitive and nanotechnologies-inspired method for early detection of misfolded Aβ in the CSF of AD patients, which meets the needs of reliability, ultrasensitivity, effectiveness, and low cost. Our approach is not only aimed at detecting Aβ strains, but also at identifying their structural features eventually exploitable for the recognition of individual AD phenotype when patients are alive (see the paragraph *Our contribution to the field and future perspectives*). 

## 5. Technological Innovations and the Future of Alzheimer’s Disease Biomarker Discovery 

In 2014, the group of Claudio Soto described a sensitive method for detecting misfolded Aβ in the CSF of patients with AD, known as Real-Time Quaking-Induced Conversion (RT-QuIC) assay. They have found that the concentration of misfolded Aβ in this fluid was in the femtomolar range; therefore, undetectable with the current diagnostic techniques. To enable its detection, they exploited the ability of the misfolded Aβ to seed the polymerization of normal Aβ in vitro by reproducing the phenomenon of protein misfolding, which occurs in vivo. This work included the analysis of 50 CSF collected from AD patients, 39 CSF from patients affected by non-degenerative neurological conditions, and 37 CSF from patients affected by other neurodegenerative diseases (non-AD). These samples were incubated with the synthetically produced Aβ_1–42_ peptide (used as a reaction substrate) and subjected to alternated cycles of incubation and shaking. Only CSF-AD samples were able to promote (*seed*) the aggregation of Aβ_1–42_, while the others did not. The aggregation of the substrate was monitored with the thioflavin T (ThT) fluorescent dye [123]. The kinetics of Aβ_1–42_ aggregation was significantly accelerated in the presence of CSF-AD, and the fluorescent signal increased earlier (*seeding effect*) than that of the other samples. Immunodepletion of misfolded Aβ from the CSF of AD patients led to a total inhibition of the Aβ_1–42_ aggregation, thus, confirming that the seeding effect was specifically triggered by misfolded Aβ present in the CSF. This was the first and only demonstration that traces of Aβ strains were present in the CSF of AD patients. Similar RT-QuIC studies have been performed in the context of other neurodegenerative diseases caused by misfolded proteins, including Parkinson’s disease (PD), dementia with Lewy bodies (DLB), and multiple system atrophy (MSA), which are caused by misfolded α-synuclein [124,125,126,127]; frontotemporal dementia (FTLD), corticobasal degeneration (CBD) and progressive supranuclear palsy (PSP) that are caused by misfolded tau [128]; and frontotemporal dementia (FTLD) and amyotrophic lateral sclerosis (ALS) that are caused by misfolded TDP-43 [129]. These studies showed that RT-QuIC could detect misfolded α-synuclein in the CSF or olfactory mucosa of patients with PD, MSA and DLB [124,125,126,130,131], misfolded tau in the CSF of patients with PiD [132], CBD and PSP [128], and misfolded TDP-43 in the CSF of patients with FTD and ALS [129].

We mentioned these studies because they led to an important discovery that can be extended to AD diagnosis. Indeed, also α-synuclein, tau, or TDP-43 can misfold and adopt a variety of aberrant structures (again referred to as *strains*) that account for the phenotypic heterogeneity of these diseases [50,133,134,135,136,137,138]. What has been observed is that each strain seemed to transfer its peculiar signature (in terms of biochemical and structural features) to the RT-QuIC substrate. Hence, the RT-QuIC reaction products acquired different biochemical, morphological, and structural properties extremely important for strain’s identification. In the light of these observations, we, along with few other research groups, have decided to couple the RT-QuIC with highly specialized biophysical and optical technologies (e.g., transmission electron microscopy, protein-NMR, cryogenic electron microscopy, Raman spectroscopy, etc.) to dissect the morphological, and structural properties of the RT-QuIC reaction products obtained from the analysis of CSF or olfactory mucosa samples of patients with PD, MSA or DLB. The results of these preliminary experiments indicated that this approach could successfully identify different α-synuclein strains allowing disease discrimination [131,139,140]. Similarly, it is possible that different misfolded Aβ can transmit their strain signature to the RT-QuIC substrate. For this reason, we decided to set-up an innovative approach for the analysis of CSF collected from patients with AD. Undoubtedly, one of the critical aspects related to the ultrasensitive analyses of these samples is represented by the limited accuracy of AD diagnosis that can lead to misinterpretation of the results.

The scenario is even more complicated by the fact that the RT-QuIC could detect peripheral Aβ strains even in subjects at the prodromal stage of AD when they are still asymptomatic, and therefore, considered ‘healthy’. Therefore, we decided to perform our analyses on samples collected from AD patients that have been extensively characterized by multiple points of view. In this regard, the Italian Ministry of Health has funded a project (concluded in 2019), led by Fabrizio Tagliavini in collaboration with other well-recognized Italian dementia centers, that thoroughly characterized (1) patients with pre-MCI, (2) patients with MCI, (3) patients with AD, (4) patients with a subjective cognitive complaint, and (5) healthy subjects from a clinical, neuropsychological, laboratory and instrumental point of view. Among the studies performed by all partners of the research group, CSF and urine were also collected at different time points for RT-QuIC assessment. These experiments are still ongoing. The objective is to evaluate the performance of the RT-QuIC and compare the results with those obtained from all the other tests. Final reaction products will be subjected to biochemical and highly specialized biophysical assessments to verify the presence of distinct properties, eventually reflecting the presence of different Aβ strains. This would consent to truly estimate whether and to what extent the RT-QuIC can identify peripheral Aβ strains in AD, pre-MCI or MCI, due to AD patients. Considering that the RT-QuIC technology has already been introduced among the clinical diagnostic criteria for prion diseases, and is currently being exploited to diagnose other neurodegenerative diseases [124,126,127,130,131,139,141,142], it is worth extending and optimizing the technology to diagnose AD.

## 6. Our Contribution to the Field of Alzheimer’s Disease Diagnosis

The urgency dictated by the need of revealing an exhaustive molecular and morphological description of RT-QuIC reaction products has inspired the ground motivations of our ERANET EuroNanoMed III SPEEDY (surface-enhanced Raman scattering with nanophotonic and biomedical amplifying systems for early diagnosis of Alzheimer’s disease pathology) project, comprising four research units from three different EU countries and supported by EU along with local funding agencies (Italian Ministry of Education, University and Research, Italian Ministry of Health, Israeli Ministry of Health, Polish National Centre For Research And Development).

The project aims to confer chemostructural information to RT-QuIC products by supporting the molecular amplification provided by RT-QuIC procedure with an optical amplification as obtained by surface-enhanced Raman spectroscopy (SERS)-based systems. Specifically, a dedicated SERS platform with advanced sensing tools, including disposable 2D-nanostructured chips and noble nanoparticles-containing optical fibers represent the main outcomes of the project, which are implemented for label-free ultradetection of misfolded Aβ, in CSF and olfactory mucosa of extensively characterized AD patients.

Raman techniques recently proved to match the requirements of a label-free characterization of the secondary structure of isolated misfolded Aβ [143] and the amyloid content from brain homogenates of PD patients [140]. Raman spectroscopy can provide information on the molecular vibrations (fingerprint of molecules) of a sample by exploiting the inelastic scattered light under visible or near-infrared excitation light. As a consequence, the noninvasive and nondestructive molecular identification and analysis of a species can be achieved. Main applications of Raman spectroscopy in biomedical research concern the investigation of different materials (biomolecules, cells, tissues, etc.) aimed at revealing their chemical composition and structure, in turn, aimed at sample discrimination, classification, and preclinical/clinical diagnosis [144,145]. However, the low efficiency of the Raman scattering process and the frequent interference by a high fluorescence background pose a limit to this technique for the detection of biomolecules, especially when examined at low concentration as typically occurs in their own biological environment. Additionally, biomolecules are characterized by the lowest Raman cross-section values, hence requiring highly sensitive systems for their detection. The weak Raman signal of biomolecular species can be compensated by taking advantage of a resonance condition, such as in resonance Raman spectroscopy (RRS) [146], by increasing the interaction between analyte molecules and excitation laser as in fiber-enhanced Raman spectroscopy (FERS) [147], by the electromagnetic amplification generated by plasmonic nanoparticles as in surface-enhanced Raman spectroscopy (SERS) [148] or by the use of single nanoantennas also pushing the spatial resolution down to the nanoscale as in tip-enhanced Raman spectroscopy (TERS) [149]. During the course of the last few years, SERS has been recognized as a technique with great potential for the analysis of biosamples.

Early achievements from some of us demonstrated the advantage of using nonspherical concave gold nanoparticles with sharp tips forming effective “hot-spots” at the junctions between multiple adjacent nanoparticles, which are at the basis of a boosted Raman signal of proteins, such as insulin and cytochrome c [150]. The amplification observed is here motivated by the formation of 2D-regular arrays of nanoparticles featuring a distribution of nanoholes for protein entrapment and detection. Afterward, preliminary results have established that SERS can offer a reliable tool for effective detection of amyloid forms by using Au-nanoparticles-decorated beads [151], as well as it was demonstrated for the first time that SERS can be exploited to identify trace proteins in a physiological environment in a quantitative and reproducible manner [152]. A low-cost SERS assay was recently introduced for sensitive detection of Aβ aggregates [153]. Here, experiments on toxic and non-toxic Aβ forms showed the possibility of using SERS for their discrimination, suggesting a high potential of the technique also for tracking Aβ during the course of its different aggregation states. At the same time, recent insights have concerned coupling noble metal nanoparticles with hollow-core optical fibers, working as effective SERS sensors for remote detection of molecular species [147]. These systems are perceived as optical tools allowing the in situ and cost-effective identification of low sampling volumes of small molecular quantities (~µg/mL) of biomarker, thanks to combined FERS and SERS effects. Furthermore, fiber-based systems can be adapted as flexible interfaces between a commercial Raman analyzer and fluid samples contained, e.g., inside multiwell plates, which is particularly attractive because of setting up a liquid biopsy assay for preclinical or clinical detection of AD.

By taking advantage of the ability of RT-QuIC to amplify different strains of Aβ with high fidelity and of SERS to reveal the presence of different misfolded structures, it would be possible to associate an early diagnosis of AD with the recognition of disease subgroup. Overall, a combined RT-QuIC/SERS technology for detecting pathological Aβ in the CSF or olfactory mucosa of patients with AD can be exploited to inform prognosis, allow monitoring of disease progression and act as an indicator of treatment effects in future clinical trials.
